# Concentrations, Distribution, Sources and Ecological Risk Assessment of Trace Elements in Soils from Wuhan, Central China

**DOI:** 10.3390/ijerph15122873

**Published:** 2018-12-14

**Authors:** Ababo Workineh Tadesse, Tekleweini Gereslassie, Qiang Xu, Xiaojun Tang, Jun Wang

**Affiliations:** 1 Key Laboratory of Aquatic Botany and Watershed Ecology, Wuhan Botanical Garden, Chinese Academy of Sciences, Wuhan 430074, China; abiyework@gmail.com (A.W.T.); tekle206@gmail.com (T.G.); 2 Sino-Africa Joint Research Center, Chinese Academy of Sciences, Wuhan 430074, China; 3 Wuhan Botanical Garden, University of Chinese Academy of Sciences, Beijing 100049, China; 4 Department of management engineering, Shangrao Vocational and Technical College, Shangrao 334109, China; xq3217@126.com; 5 Wuhan Britain-China School, Wuhan Foreign Languages School, Wuhan, 430022 China; Tangxj2002@163.com

**Keywords:** trace elements, concentration, microwave digester, Wuhan, soil contamination, ecological risk

## Abstract

This study aimed to determine the concentration levels, potential sources and ecological risks of eleven trace elements, namely Cr, Fe, Co, Ni, Cu, As, Sb, Cd, Zn, Hg and Pb, in the soil from Huangpi district, Wuhan, Central China. Soil samples were collected from eighteen sites at soil depths of 1–10 and 10–20 cm and analyzed using Inductively Coupled Plasma-Mass Spectrometer ICP-MS (Thermo X SERIES 2, Scientific and Innovative Technology Co. Ltd., Beijing, China). The recorded mean concentration of the elements were in a decreasing order of Fe > Co > Cr > Ni > Pb > Cu > As > Cd > Sb > Zn > Hg. The mean concentration of trace elements, soil pH and total organic carbon (TOC) were higher at a soil depth of 1–10 cm. The obtained mean concentration of Cr, Co, As, Cd, Ni, Cu, Hg and Pb were above the soil background values of Wuhan and Hubei Province. The mean concentration values of Co, Ni and Cd, exceeded the recommended FAO (Food and Agriculture Organization)/ISRIC (International Soil Reference and Information Centre) (2004) and WHO/FAO (2001) values. Pearson’s correlation analysis illustrated that there was a strong and significant correlation between trace elements, whereas, a weak positive and negative correlation between elements and soil properties (pH and TOC). The principal component analysis (PCA) and cluster analysis (CA) result indicated that the concentration of trace elements in Huangpi soil were originated from anthropogenic sources. Potential ecological risk index (RI) of this study revealed that there is a high ecological risk of trace elements in the soil. Enrichment factor (EF) and geo-accumulation index (*I**_geo_***) of trace elements for this study indicated that the study area is strongly contaminated with Cd and Co. Generally, the finding of this research showed that Huangpi soil is contaminated.

## 1. Introduction

Trace elements are identified as a dangerous group of environmental pollutants, due to their persistence, non-degradability and toxicity to living organisms [1,2]. Unlike many organic pollutants, which eventually degrade to carbon dioxide and water, trace elements tend to accumulate in the environment, especially in soil and sediments [3]. Trace elements have specific gravity greater than 5 g/cm^3^ and they include elements such as Cd, Zn, Pb, Hg, Sb, Cr, Co, As, Ni Cu, Mo, and Mg [4,5]. Trace elements like Cd, Cr, Pb, Hg, Ni and As have been listed as the most dangerous elements and priority control pollutants by the United States Environmental Protection Agency (USEPA) [6,7,8]. Trace elements can originate from natural sources (e.g., parent materials, weathering of rocks, volcanic eruptions and soil erosion) and anthropogenic sources (e.g., industrialization, urbanization, vehicular emissions, mining activities, smelting, burning of fossil fuels and agricultural inputs such as fertilizers, pesticides, herbicides and fungicides [9,10]. However, currently anthropogenic activities significantly accelerate the accumulation of trace elements in the environment [11,12].

Soil is one of the environmental compartments which plays an important role in plant growth, development and other ecosystem services [8,13]. On the other hand, it also serves as a sink for different environmental pollutants like toxic trace elements [14,15,16]. Several studies from different countries point out the toxic effects of trace elements on soil, plants and animals [1,3,12]. Excessive accumulation of trace elements in soil can reduce soil microorganism levels which results in soil quality degradation [16,17], and reduction of agricultural productivity [3,15,18]. It has been reported that foodstuffs and domestic feeds in Asian countries are severely contaminated with trace metals [19]. According to China national census of pollution report, more than 1.5 million sites in China have been exposed to toxic trace elements, 20 million hectares of agricultural lands have been polluted [20] and over 12 million tons of grains are contaminated by toxic elements per year [7]. About 1.5 × 10^5^ km^2^ of cultivated lands is polluted by Cd [21]. In addition to the above reports, a survey conducted in 2014 indicated that 16.1% of the sampled lands were contaminated by Hg, As and Pb [6].

Plants not only absorb essential nutrients, they also absorb toxic elements from the environmental compartments [22], which results in a bio-accumulation effect [14,23]. Consequently, consumption of contaminated plants and animals can cause health problems [24]. Particularly, Cd is amongst trace metals that increased international concern due its carcinogenic effect [21]. The adverse effects of toxic trace elements on human beings have been reported from different countries of the world [24,25]. For example: As causes dermal lesions, skin cancer, peripheral vascular diseases and peripheral neuropathy [26,27], Cd can result in kidney dysfunction, hypertension, lung cancer, bone fractures, prostatic hyperplasia and adenocarcinomas, Cu can cause Alzheimer’s, prion disease [28,29], and Pb can affect the endocrine system, immune system, skeletal, circulatory system and nervous system [30].

Due to the rapid economic growth in China, there are heterogeneous anthropogenic activities which increase the accumulation of trace elements in the environment [31]. Agricultural inputs (fertilizers, herbicides, pesticides and fungicides), the use of wastewater for irrigation, urbanization, industrialization and construction of road networks are the major anthropogenic activities in Wuhan, Hubei Province [32,33,34]. Huangpi is one of the districts in Wuhan, in which the above anthropogenic activities are commonly observed [20]. However, there is lack of data and research works on the status of trace elements in Huangpi district. Previously conducted studies in Wuhan were focused only on few trace elements such as Cd, Cr, Pb Ni, As, Cu and Hg. Therefore, it’s important to investigate the status of the above listed and other additional trace elements like Co, Sb, Fe, Zn in the soil of Huangpi district. Thus, the objectives of this study were: (1) to determine the concentration level of trace elements, (2) to identify the potential sources of trace elements, (3) to evaluate pollution status and ecological risks of trace elements in the soil and (4) to determine the effect of soil pH and total organic matter on the concentration of trace elements.

## 2. Materials and Methods

### 2.1. Study Area

**Figure 1:**
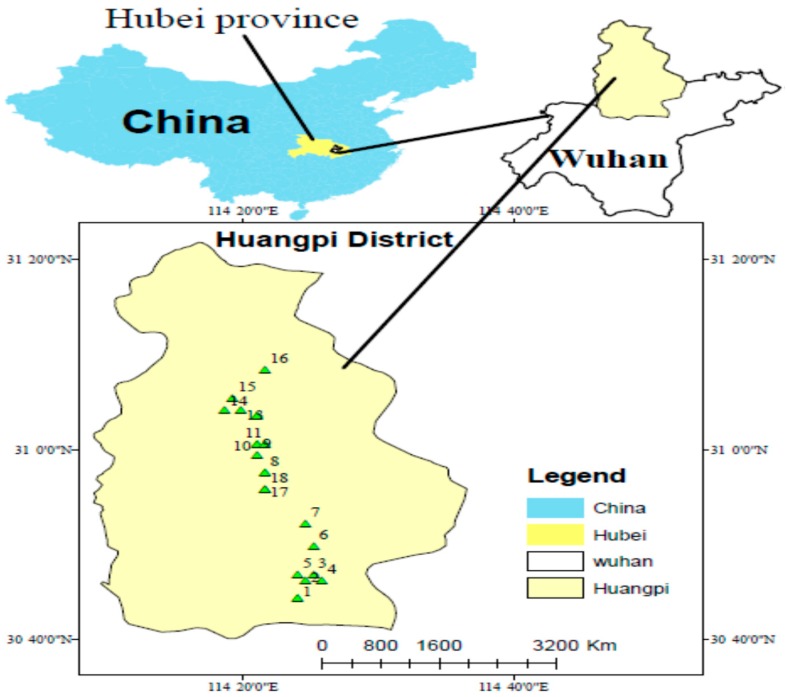
Study area. (The number (1–18) represents sampling sites: S1 (Tangjiawan), S2 (Fengdouhu) S3 (Erpaiqu), S4 (Changdi), S5 (Zhujiashan), S6 (Tujiadun), S7 (Zhulinyuan). S8 (Zhoujiawan), S9 (Lishuwan), S10 (Xinyang), S11 (Leqianwan), S12 (Bomogang), S13 (Hanjiafan), S14 (Wanjiatian), S15 (Hongguanshanxiawan), S16 (Dujiatian), S17 (Tianjiaxiaowan), S18 (Tianjiaxiaowan).

Soil samples were collected from Huangpi district, Wuhan, Central China. Huangpi is one of thirteen districts of Wuhan, located on northern outskirts of Wuhan between 30°52’30’’ N and 114°22’30’’ E (Figure 1). The area of the district is about 2261 km^2^, with a population of 1,107,565 [31]. From the total area of the land 56.12%, 18.29%, 0.22%, 7.09%, 0.21%, 23% and 4.08% are covered by cultivated land, forest land, grassland, settlements and industrial sites, land for transport, water area and unused land, respectively [35].

### 2.2. Sample Collection and Pretreatment

Soil samples were collected from eighteen sampling sites of different land use types (barren land, farmland, paddy field, plastic greenhouse) at soil depths of 1–10 and 10–20 cm. The sample sites were located using a global positioning system (GPS). For each site, three replicates were taken to make up a composite soil. The collected samples were packed in polyethylene containers, labeled and transported to the laboratory for analysis. All samples were dried using a benchtop lab vacuum freeze dryer (Xinzhi Biotechnology Co., Ltd., Ningbo, China) at −40 °C for 24 h, the samples were ground, sieved through 0.6 mm mesh nylon sieve and stored in a refrigerator at −20 °C for further analysis [12,36].

### 2.3. Sample Extraction and Analysis

About 0.1–0.15 g of soil samples were measured using a digital analytical balance (METTLER TOLEDO, Columbus, OH, USA), placed into Teflon vessels, digested with 4 mL of nitric acid (HNO_3_, 63%), 2 mL of hydrogen fluoride (HF ≥ 40%) and perchloric acid (HClO_4_ 70–72%) using a microwave digester (ETHOS ONE, Milestone, Leutkirch im Allgau, Germany). The digester was operated for 2 h at a controlled pressure, temperature and output power according to [37]. The digested samples were heated on the heating plate at a temperature of 135 °C for 2 h and cooled to room temperature. Then the final volume was topped up to 50 mL using double distilled water and filtered using 0.22 um membrane filter paper for analysis [22,36] and the filtered samples were stored in plastic bottles at −4 °C to minimize volatilization and biodegradation [38]. Finally, the samples were analyzed using ICP-MS (Thermo XSERIES2, Beijing, Scientific and Innovative Technology Co. Ltd., Beijing, China). In addition, selected soil properties (soil pH and TOC) were measured. Soil pH was measured according to [22], using a pH meter (METTLER TOLEDO) and the total organic carbon (TOC) of the soil was measured by [39] method using a TOC analyzer (Elementar GmbH, Langenselbold, Germany).

### 2.4. Standard Preparations and Calibration Curves

All chemicals and reagents used were analytical grades with purity of 99%. Five working solutions with concentrations of 0.1, 0.5, 1, 5, 10 and 20 µg/L were prepared from the stock solutions of 1000 µg/mL using double distilled water with 10% nitric acid (HNO_3_, 63%). The working solutions were analyzed before running samples to check the accuracy and reliability of the instrument. Calibration curves produced for all elements indicated that the obtained curves had r^2^ values greater than 0.994.

### 2.5. Statistical Analysis

Statistical Package for Social Sciences (SPSS) version 20 (IBM, Armonk, NY, USA) and Microsoft Offices Excel 2013 (Microsoft Corporation, Albuquerque, NM, United States) were used to analyze the data. Pearson’s correlation coefficient was used to analyze the relationships between elements, soil pH and TOC. The potential sources of trace elements were identified using factor analysis (PCA and cluster analyses).

### 2.6. Quality Analysis and Quality Control

To ensure the quality of the experiment all reagents and chemicals used were analytical-reagent grades. All plastic containers were soaked with 10% HNO_3_ (63%) for 12 h and washed three times with double distilled water to remove other contaminants, dried in oven at 60–65 °C for 24 h. The relative standard deviations (RSD) of three consecutive measurements of the standard solutions were used to determine the precision of ICP-MS. The obtained percentage of relative standard deviation (% RSD) was less than 10% indicating a good precision of the instrument. The recovery value of elements ranged between (90–104%).

### 2.7. Methods of Evaluating Contamination Level and Ecological Risk of Trace Elements

#### 2.7.1. Potential Ecological Risk Index (RI)

Potential ecological risk index helps to evaluate the pollution of trace elements in the soil [40]. It is the sum of the ecological risk factor of a single element in the sample [41]. The following formulas given by [41] were used to calculate potential ecological risk indices of the elements.

(1) $$C_{f}^{i}=C_{S}^{i}/C_{R}^{i}$$

(2) $$Cd=\Sigma_{f}^{i}C_{f}^{i}$$

(3) $$E_{f}^{i}=C_{f}^{i}*T_{f}^{i}$$

(4) $$RI=\Sigma_{f}^{i}/E_{f}^{i}$$

where *C^i^_f_* is the contamination factor for trace elements; *C^i^_S_* is the measured concentrations value of elements in the soil; *C*^i^_R_ is the background reference values of trace elements in the soil; *C_d_* is a degree of contamination; *E^i^_f_* is the ecological risk index of a single element; *T^i^_f_* is the toxicity coefficient of an element and *RI* represents the total potential risk index of elements [40] (Table 1).

Due to the absence of soil background reference values of trace elements in Huangpi district, Wuhan soil background values were used as a reference: Cr (90), Ni (40), Cu (35), Zn (100), As (15), Cd (0.20), Hg (0.15), Pb (35) mg/kg [42] and Hubei Province soil background values were used for Co (15.4) [32], Sb (1.65 mg/kg) [43] and Fe (29,400 mg/kg) [40]. *T**^i^_f_*** values given by [41] were Cr = 2, As = 10, Cd = 30, Ni = Cu = Pb =5, Zn =1, Hg = 40 and Co = 2, Fe = 0 and Sb = 15.

#### 2.7.2. Enrichment Factor (EF)

Enrichment factor (*EF*) was evaluated to determine the degree of anthropogenic factors on trace elements accumulation in the soil. It reflects the disturbance degree of human activities on the natural environment [44]. *EF* is calculated as follows:

(5) $$EF=\frac{\left(\frac{C_{i}}{C_{ref}}\right)sample}{\left(\frac{B_{i}}{B_{ref}}\right)background}$$

where *C_i_* is the concentration of trace elements in the sample; *C_ref_* is the concentration of reference element in the sample; *B_i_* is the background value of interest element and *B_ref_* is the background value of reference elements in the study area. Fe was selected as the reference element for this study because Fe is a major sorbent and it is a quasi-conservative tracer of natural elements in fluvial and coastal sediments [12]. Wuhan soil background was used as the reference. The following EF class were given for elements; *EF* < 2, 2 ≤ *EF* < 5, 5 ≤ *EF* < 20, 20 ≤ *EF* < 40 and *EF* ≥ 40 indicated low, moderate, significant, very high and extremely high enrichment factor, respectively.

#### 2.7.3. Geo-accumulation Index (*I_geo_*)

The geo-accumulation indexes (*I_geo_*) of elements were calculated to identify the degree of contamination and to compare it with the pre-industrial level [45]. This method classifies the pollution level of elements in terms of seven enrichment classes. It is calculated using the following formula:

(6) $$I_{\mathrm{geo}}={\mathrm{Log}}_{2}\;\frac{C_{n}}{1.5\;B_{n}}$$

where *I_geo_* is the geo-accumulation index, *C_n_* is the measured concentration of an element (mg/kg) in the sample, *B_n_* is the geochemical background value of an element (mg/kg) and 1.5 is the factor used for lithological variations of elements.

Seven classes and contamination intensity are given for *I_geo_*: *I_geo_* ≤ 0 (uncontaminated), 0 < *I**_geo_*** ≤ 1 (uncontaminated to moderately contaminated), 1 < *I**_geo_*** ≤ 2 (moderately contaminated), 2 < *I**_geo_*** ≤ 3 (moderately to strongly contaminated), 3 < *I**_geo_*** ≤ 4 (strongly contaminated), 4 < *I**_geo_*** ≤ 5 (strongly to extremely contaminated) and *I**_geo_*** > 5 (extremely contaminated) [45].

## 3. Result and Discussion

### 3.1. Spatial Distribution of Selected Soil Properties (pH and TOC)

**Figure 2:**
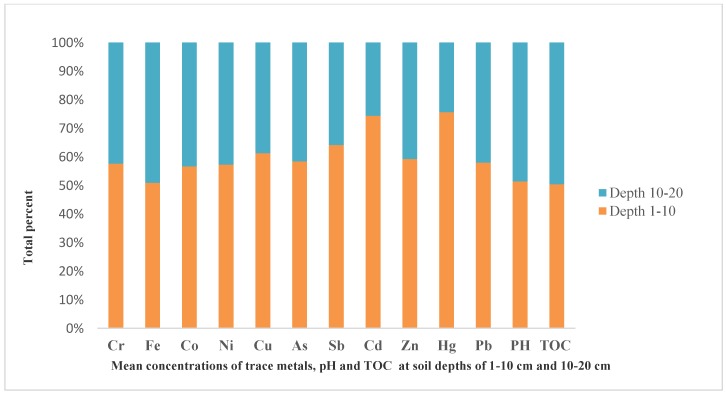
The mean concentrations of trace elements and selected soil properties (pH and TOC) at soil depth of (1–10 and 10–20 cm).

It has been reported that soil properties such as soil pH and total organic carbon (TOC) are the most important factors that influence cation mobility and regulate the solubility of trace elements in the soil. The obtained mean result of soil pH and TOC for this study were presented in Table 2. As illustrated, the mean soil pH results ranged from 4.20–6.87 with a mean value of 5.71. The comparison between individual samples (Table 3) indicated the soil pH ranged from 4.12–8.07 with an average of 5.87 at the soil depth of 1–10 cm and 4.28–6.52 with an average of 5.55 at the soil depth of 10–20 cm. The highest soil pH value (8.07) was recorded in the greenhouse soil from Changdi site at a soil depth of 1–10 cm. The main reason for this might be connected with relatively low precipitation amount and less leaching of base-forming cation in the green house. The mean comparison between the two depths indicated that the soil pH at 1–10 cm (5.87) is higher than the soil depth of 10–20 cm (5.55) (Figure 2). The result is in line with the findings of [46,47], whereas the result is different from the findings of [48]. According to the classification of soil pH grade, the pH value of (<5) indicated slightly acidic, (5–6.5) mildly acidic, (6.5–7.5) neutral, (7.5–8.5) mildly alkaline and (>8.5) indicated strongly alkaline [49]. According to these classifications, Huangpi soil is classified as slightly acidic, mildly acidic, neutral and mildly alkaline.

The average total organic carbon of soil ranged from 0.65–2.41 with the mean value of 1.71. The highest mean value of TOC (2.41) was recorded in Dujiatian site (farmland), whereas the lowest TOC (0.65) was recorded from barren land at the Zhoujiawan site. TOC ranged from 0.63–2.60 with a mean value of 1.74 at soil depth of 1–10 cm and 0.66–2.71 with a mean value of 1.71 at the soil depth of 10–20 cm. The highest mean was recorded at the soil depth of 1–10 cm (Figure 2). A similar result was reported by [47,50]. Numerous studies indicated that TOC of soil decreases with soil depth [50,51]. The main reason for this might be biological activity in the top layer of soil [51].

### 3.2. Concentrations and Distribution of Trace Elements

Eleven trace elements in Huangpi district soil were studied. The results of descriptive statistics; mean, maximum, minimum, standard deviation and Skewness were presented in Table 2 and individual results of all samples and mean for both depths were presented in Table 3 and Figure 2, respectively. The results indicated that the concentrations of the elements showed variation between samples and within samples at different soil depths. All elements except As, Sb, Hg and Cd were detected in all samples at soil depths of 1–10 and 10–20 cm with detection frequency of 100% for Cr, Fe, Co, Ni, Cu, Zn and Pb; and 72%, 89%, 78% and 42% for As, Sb, Cd, Hg respectively. The mean concentration of trace elements at soil depth of 1–10 cm were in decreasing order of Fe > Co > Cr > Ni > Pb > Cu > Cd > As > Zn > Sb > Hg, whereas, Fe > Co > Cr > Ni> Pb > Cu > As > Cd > Zn > Sb > Hg at soil depth 10–20 cm (Figure 2).The highest mean concentration values for all elements were recorded at a soil depth of 1–10 cm. The results of this study was in line with those of [52,53,54]. According to Camobreco [55] the highest accumulation of trace elements in the surface layer of the soil might be due to a high sorption capacity of trace elements which results from soil chemical reactions between soil solid phases, including silicate clays, hydroxides and oxides of elements. Another study by Rahaman [53] also indicated that trace elements were found abundantly in the surface layer and the value decreased with an increase in soil depth with few exceptions. Converse to the results obtained in this study, the report by [56] from Kenya, indicated that subsurface soil accumulates high concentration of trace elements than surface soil due to soil leaching.

The highest mean of Cr was recorded in the sample from Dujiatian (farmland), whereas the lowest was recorded at Tangjiawan (barren land). The obtained mean result of Cr (140.10 mg/kg) was higher than the soil background value of Wuhan (90 mg/kg) and Hubei Province (86 mg/kg), but less than soil background of China (200 mg/kg). In comparison to the permissible limit of FAO (Food and Agriculture Organization)/ISRIC (International Soil Reference and Information Centre) (2004) (250 mg/kg) and USEPA (1983) (1000 mg/kg) the mean concentration value of Cr (140.10 mg/kg) was lower. However, the individual mean result of single site indicated that the sample from Dujiatian tea farmland (321.73 mg/kg) was above the permissible limit of FAO/ISRIC (2004). As compared to the other findings in Wuhan, the obtained mean value of Cr for this study was higher than the finding of [32] (85 mg/kg), but less than the finding by [33] (152.78 mg/kg). In comparison to the other studies from other places, the mean concentration of Cr for this study was higher than the findings from Cuba (85.9 mg/kg) [59], Bangladesh (53.7, 34.2 mg/kg) [60], India (8.01 mg/kg) [61], Tanzania (7.68 mg/kg) [10], Brazil (20.61 mg/kg) [62], Pakistan (5.86 mg/kg) [63], Iran (48.08, 53.21 mg/kg) [64], along Chao River in China (118 mg/kg) [65] and Northern Pakistan (29.94 mg/kg) [66].

The maximum mean value of Fe (55,398.01 mg/kg) was recorded at Dujiatian farmland site, whereas the lowest value was recorded at Tangjiawan site in the soil from a barren land. The recorded mean value of Fe was below the soil background value of Hubei Province and China (29,400 mg/kg). As compared to the other studies, the mean concentration of Fe (27,304.9 mg/kg) from this study was higher than a study from Bangladesh [11] (1800 mg/kg).The highest concentration of Co (55,580.36 mg/kg was obtained at the Dujiatian site (farmland), whereas, the lowest value (4355 mg/kg) was recorded at Tangjiawan site (barren land). The obtained mean Co was higher than the soil background value of Wuhan, Hubei Province and China (Table 2). The mean concentration value of Co (22,656.94 mg/kg) was above the permissible limit of FAO/ISRIC (2004) and WHO/FAO (2001) (Table 2). Compared to other findings the mean concentration of Co was higher than the study in Cuba (9.16 mg/kg) [59], Brazil (7.44 mg/kg) [62], Pakistan (7.56 mg/kg) [63], Wuhan (China) (16 mg/kg) [32] and (16.37 mg/kg) [33], Iran (38.5, 16.51 mg/kg) [64], Chao River China (17.5 mg/kg) [65] and Northern Pakistan (36.76 mg/kg) [66].

The recorded mean concentration of Ni (117.80 mg/kg) was higher than the soil background value of Wuhan, Hubei province and China. In addition, the mean value of Ni (117.80 mg/kg) was above the permissible limit of FAO/ISRIC (2004) (100 mg/kg), WHO /FAO (2001), whereas below the permissible limit of USEPA (1983) (500 mg/kg). The highest value of Cu was recorded at Dujiatian (farmland), whereas the lowest was obtained from the soil of Tangjiawan site (grassland soil). According to the obtained result, the mean concentration of Cu recorded was below the permissible limit of FAO/ISRIC (2004), WHO/FAO (2001), USEPA (1983) and China (Table 2). However, the individual result of each site indicated that the concentration of Cu at Dujiatian site exceeded the permissible limit of FAO/ISRIC (2004), WHO/FAO (2001) and USEPA (1983). As compared to the other studies the mean concentration value of Cu (60.73 mg/kg)was less than the finding in Brazil (111.54 mg/kg) [62], Wuhan, China (60.85 mg/kg) [33], Hubei Province, China (386mg/kg), Democratic Republic of Congo (10,320 mg/kg) [67], Iran (100.84 mg/kg) [64], but the result was higher than the findings in soil from Cuba (43.10 mg/kg) [59], Bangladesh (20.6 mg/kg) [11], India (52.72 mg/kg) [61], Tanzania (5.62 mg/kg) [10], Pakistan (18.12 mg/kg) [63], Northern Pakistan (35.28 mg/kg) [66] and in soil along the Chao River in China (46.5 mg/kg) [65].

The highest value of As was recorded at Zhulinyuan site from a barren land, whereas the lowest was recorded at Hanjiafan from a paddy field. The mean concentration result of As was higher than soil background values of Wuhan and Hubei province (Table 2). The average concentration of As recorded was below the permissible limit of USEPA (1983). However, the individual result of each site indicated that the concentrations of As in samples from Fengdouhu, Changdi, Zhulinyuan, Zhoujiawan and Zhujiashan sites were above the soil background value of China (30 mg/kg). The mean comparison of As concentration of this study with the other studies are presented in Table 4.

The obtained mean concentrations of Sb (0.58 mg/kg) was lower than the soil background value of Hubei (1.65 mg/kg) and China (10 mg/kg). The mean result of Sb in this study was lower than the finding from Brazil (13.81 mg/kg) [62], whereas higher than the finding from Iran (0.22, 0.21 mg/kg) [64]. The maximum value of Cd was recorded in the sample from Zhujiashan from a greenhouse, whereas the lowest was recorded at Tangjiawan from barren land. The mean concentration of Cd (15.44 mg/kg) was above the soil background values of Wuhan, Hubei Province and China. In addition the concentration of Cd in this study passes the permissible limit of FAO/ISRIC (2004), WHO/FAO (2001) USEPA (1983). In comparison to the other studies the concentration of Cd for this study was lower than the finding of [62] (13.81 mg/kg) and [67] (49 mg/kg) (Table 4). 

The mean concentration of Zn for this was lower than the soil background value of Wuhan, Hubei and China. As compared to the permissible limit of FAO/ISRIC (2004) (500 mg/kg), WHO/FAO (2001) (300 mg/kg), USEPA (1983) (500 mg/kg) and China 250 mg/kg the mean concentration of Zn in this study was lower.

The mean result of Hg was higher than the soil background value of Wuhan, Hubei Province and China (Table 2), but below, however, the permissible limits of FAO/ISRIC (2004), WHO/FAO (2001) and USEPA (1983). However, the result of the individual site indicated that sample from Dujiatian farm (1.13 mg/kg) exceeded the permissible limit of WHO/FAO (2001) (1 mg/kg). In comparison to the other studies, the obtained mean concentration of Hg was lower than the finding of in soil from Cuba [59], Bangladesh [60], Brazil [62], Iran [64] and soil along the Chao River in China [65]. 

The mean concentration of Pb was below the recommended value of FAO/ISRIC (2004) and USEPA (1983), however, above the permissible limit of WHO/FAO (2001) (Table 2). The concentration of Pb in samples from Changdi, Zhujiashan, Zhulinyuan, Lishuwan, Xinyang, Bomogang and Tianjiaxiaowan site exceeded the soil background value of China (80 mg/kg) (Table 2)**.**

### 3.3. Relationships of Trace Elements and Selected Soil Properties (pH and TOC)

Pearson’s correlation was applied to analyse the relationships between trace elements, soil pH and TOC. The obtained result indicated that there was a significant positive correlation between trace elements at a significant level of 0.01 and 0.05 (2-tailed) whereas, there was a negative and weak correlation between trace elements and selected soil propertis (pH and TOC). Strong positive correlations of Cr with Fe, Co, Ni, Cu, and Hg, Fe with Co, Ni, Cu and Hg, Co with Ni, Cu, and Hg, Cu with Hg, As with Cd, Sb with Cd, Zn and Pb and Zn with Pb were observed at a significance level of 0.01 and 0.05 (Table 5). Moderate and weak correlations between trace elements were also obtained (Table 5). The relationships between trace elements and soil properties indicated that soil pH has a weak positive correlation with Fe, Ni, Cu, As, Sb, Cd and Pb, whereas a negative correlation with Cr, Co, Zn and Hg. The same finding was reported by [47], for Cr, As, Hg and Pb from Guangzhou (China). TOC had a negative correlation with Co, As, Sb, Cd and Hg, and a weak correlation with Cr, Fe, Ni, Cu, Zn and Hg. The same result was reported on Cd by [69]. Weak and negative correlations were observed between soil pH and TOC. A significant positive correlation among elements indicates a common origin. Moderate correlations among elements show those elements share a common source but they are not totally from the same sources, and a weak correlation among those elements indicates they have different origins. A negative correlation among elements and soil properties indicates no influence of soil properties (pH and TOC) on the distribution and concentration of trace elements in the soil.

### 3.4. Factor Analysis

Principal Component Analysis (PCA) is a dimensional reduction tool that is used to reduce large and complex data to a small set of variables which makes it easy for interpretation [70]. It is used to identify correlated variables having common sources [71]. PCA result for trace elements is presented in Table 6. The significant principal component is selected based on the basis of Varimax rotation of Kaiser Criteria with an eigenvalue of greater than 1 [9]. The result indicated that eigenvalues greater than 1 gave a total cumulative value of 86.029%. The variables were correlated with two principal components.

Two-component factors were extracted and the result indicated that the first component with an eigenvalue of 7.167 and with a variance of 65.125% was highly correlated with the high loadings of Cr, Fe, Co, Ni, Cu and Hg (Table 6). According to PCA l values, high and strong positive loading of the elements Cr, Fe, Co, Ni and Cu was connected to anthropogenic factors. The samples were collected from different agricultural fields (paddy field, farm land, vegetable field, plastic greenhouse and barren land). Comparison between fields indicated that the highest value of Cr, Fe, Co, Ni, Cu and Hg was recorded in farmland field. This indicated that the potential sources for these elements might be associated with agricultural inputs (fertilizers, pesticides, herbicides and fungicides) and use of wastewater for irrigation. Different studies have indicated that phosphate fertilizers are highly used in China [6]. A previous study [6] reported that phosphate fertilizers are the main sources of trace elements in the environment. PCA 2 gave eigenvalue of 2.296 and % variance of 20.877 with high loading of As, Sb, Cd Zn and Pb. The PCA 2 might be connected with both anthropogenic and natural factors (parent minerals, weathering processes) and different point and nonpoint sources (application fertilizer, mining, industrial discharge, using wastewater for irrigation). A study by [19] indicated that As might have originated from parent minerals.

### 3.5. Cluster Analysis

**Figure 3:**
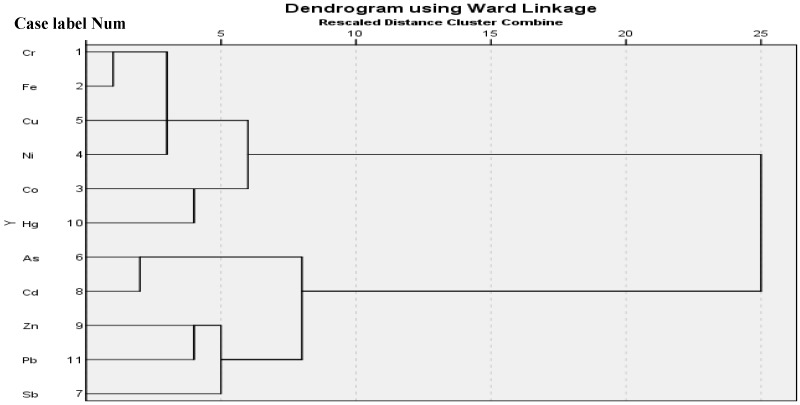
Dendrogram of hierarchal cluster analysis for trace elements in soil using Ward methods.

Cluster Analysis (CA) is a method used to group data according to their similarity [19]. Hierarchical Cluster Analysis (HCA) was applied based on the Wards method and Euclidean distance methods [9]. The HCA result (Figure 3) indicated that elements were clustered into two major clusters. The first cluster includes Cr, Fe, Cu, Ni, Co and Hg. The second cluster includes As, Cd, Zn, Pb and Sb. HCA indicated that there is a close cluster between Cr and Fe, Ni and Cu, Co and Hg, As and Cd and Zn and Pb. This implied that those elements were from the same sources. Pearson’s correlation analysis also pointed out that those elements had a strong positive correlation which suggested that the elements have the same common origin. The moderate cluster between elements (Figure 3) indicated that these elements share similar sources.

### 3.6. Contamination Level and Ecological Risk of Trace Elements

Contamination factor, degree of contamination, ecological risk factor, potential ecological risk index and enrichment factor and the geo-accumulation index of trace elements were evaluated and the results presented in Table 7. 

The obtained result of (*C^i^_f_*) indicated that Co > Cd > Ni > Pb > Cu > Cr > As > Hg > Fe > Sb > Zn. According to the obtained value of (*C^i^_f_*), the contamination level of trace elements are grouped as low contamination from Zn (0.03), Fe (0.93) and Sb (0.35), moderate contamination from Cr, Ni, Cu, As, Hg and Pb and a high contamination of soil from Co and Cd. The degree of contamination (*C_d_*) for elements for this the study was 1560.2, which indicates a high degree of soil contamination. Ecological risk factors (*E^i^_f_*) for single elements were in the decreasing order of Co > Cd > Hg > Ni > Pb > As > Cu > Sb > Cr > Zn > Fe. According to the classification given for ecological risk factor for single elements in Table 1, Cr, Ni, Cu, As, Zn, Pb, Fe and Sb had low-risk factors, Hg had a moderate risk factor and Cd and Co had an extremely high risk factors. Thus, Cd, Hg and Co can cause high risk to human and environment in Huangpi district. The same result was reported on Cd and Hg from East Dongting and Honghu Lake in Hunan Province, China [37]. The potential risk index (*RI*) for this study was 5352.03, which indicated a high ecological risk due to these trace elements. The obtained enrichment factor of elements revealed that soil is enriched with Co and Cd, moderately enriched with Ni, Cu, Hg and Pb; and less enriched with Cr, Fe As and Sb (Table 7). This indicated that there is a high rate of anthropogenic disturbance in Huangpi soil. Extremely high enrichment for Cd was also reported by [72] and [73]. The obtained *I_geo_* values of the elements in soil were in decreasing order of Co > Cd > Ni >Cu > Pb > Cr > As > Hg > Fe > Sb > Zn. According to the seven classes proposed by [45], the obtained *I_geo_* result revealed that the soils under study were extremely contaminated with Cd and Co, moderately contaminated with Cr, Ni, Cu and Pb and less contaminated with As, Zn, Hg, Fe and Sb. The obtained *I_geo_* result for Cd was in line with those of [72,74,75].

## 4. Conclusions

Eleven trace elements in soil from Huangpi district were studied. All trace elements except As, Sb, Hg and Cd were detected in all samples at both soil depths. The obtained mean concentration of Cr, Ni, Cu, As, Cd, Pb were above the soil background value of Wuhan and Hubei Province. The mean concentration values of Cd and Co exceed FAO/ISRIC (2004), WHO/FAO (2001) and USEPA (1983) recommended values of trace elements in soil. The mean concentration of Ni was above the permissible limit of FAO/ISRIC (2004), WHO/FAO (2001), but less than the permissible limit of USEPA (1983). Pearson’s correlation result indicated that there was a significant positive correlation among trace elements, whereas, weak and negative correlations between trace elements and soil properties (pH and TOC). PCA, HCA and EF of the soil indicated that anthropogenic factors are the major sources of trace elements in Huangpi soil. The result of contamination factor (*C^i^_f_*) for trace elements were in decreasing order of Co > Cd > Ni > Pb > Cu > Cr > As > Hg > Fe > Sb > Zn. The obtained *C_d_* values indicated that there is high degree of soil contamination. The result of ecological risk factor (*E^i^_f_*) of elements were in the decreasing order of Co > Cd > Hg > Ni > Pb > As > Cu > Sb > Cr > Zn > Fe. Potential ecological risk index (*RI*) result for the studied soil (*RI* > 600) indicated that there is a high ecological risk of elements in the study area. Enrichment factor (EF) showed that there is extremely high enrichment from Cd and Co, moderate from Ni, Cu, Hg and Pb, and low from Cr, Fe, As, Sb and Zn. The geo accumulation index (*I**_geo_***) results point out that the study area is highly contaminated with Cd and Co. From the obtained result it’s concluded that Huangpi soil is contaminated with trace elements. Therefore, more attention should be given and remediation action should be set to minimize the concentration and ecological impacts of trace elements in the study area. Further research should be conducted on human health impact of trace element in Huangpi soil.

## Figures and Tables

**Table 1:** The standard given for the contamination factor, degree of contamination, ecological risk factor and risk index of elements.

*C^i^_f_*	Contamination Level	*C_d_* Class	Degree Contamination Level	*E^i^_f_*	Pollution Degree	*RI*	Risk Degree
*C^i^_f_* < 1	Low contamination factor	*C_d_* < 8	Low	*E^i^_f_* < 40	Low risk	*RI* < 150	Low ecological risk
1 ≤ *C^i^_f_* < 3	Moderate contamination	8 ≤ *C_d_* < 16	Moderate	40 ≤ *E^i^_f_* ≤ 80	Moderate risk	150 ≤ *RI* < 300	Moderate ecological risk
3 ≤ *C^i^_f_* < 6	Considerable contamination factor	16 ≤ *C_d_* < 32	Considerable	80 ≤ *E^i^_f_* <160	Considerable risk	300 ≤ *RI* < 600	Considerable ecological risk
*C^i^_f_* ≥ 6	Very high contamination	*C_d_* ≥ 32	Very high	160 ≤ *E^i^_f_* < 320	high risk	*RI* > 600	Very strong
				*E^i^_f_* ≥ 320	Extremely high		

Source: [41].

**Table 2:** Descriptive statistics of trace elements (mg/kg) and selected soil properties (pH and TOC) of soil from Huangpi district and background value of Wuhan, Hubei and China.

Elements	Min	Max	Mean	SD	Skewness	(a)	(b)	(c)	(d)	(e)	(f)
Cr	66.56	321.73	140.1	58.84	1.795	90	86	200	250	-	1000
Fe	13,583.04	55,398.01	27,304.9	10,705.1	1.148	-	29,400	29,400	-	-	-
Co	7,244.46	5,4621.91	22,656.94	10,317.8	1.578	-	15.4	40	50	50	-
Ni	51.18	210.63	117.8	50.93	0.701	40	37.3	50	100	100	500
Cu	26.09	139.98	60.73	30.06	1.197	35	30.7	100	100	100	100
As	ND	47.58	15.58	17.68	0.826	15	12.3	30	-	-	75
Sb	ND	1.54	0.58	0.42	0.662	-	1.65	10	-	-	-
Cd	0.07	77.62	15.44	23.84	1.69	0.2	0.172	0.5	5	3	0.7
Zn	1.53	4.81	3.32	0.94	−0.544	100	83.6	250	500	300	300
Hg	ND	1.13	0.15	0.26	3.458	0.15	0.08	0.7	-	2	1
Pb	38.81	117.9	74.16	20.22	0.395	35	26.7	80	150	50	200
pH	4.2	6.87	5.71	0.73	−0.543	-	6.5	-	-	-	-
TOC	0.65	2.41	1.72	0.47	−0.815	-	-	-	-	-	-

ND: Not detected; Max = Maximum; Min=minimum; SD = Standard deviation. (a), Wuhan soil background value [42], (b), Hubei province soil background [37], (c), china soil background value [27], (d), Food and Agriculture Organization (FAO)/International Soil Reference and Information Centre (ISRIC) (2004) [57] (e), World Health Organization (WHO)/Food and Agricultural Organization (FAO) (2001) [12], (f), United States and Environmental Protection Agency (USEPA) (1983) [58].

**Table 3:** The concentration of trace elements (mg/kg), soil pH and TOC for each sample at soil depths of (1–10 and 10–20 cm).

S	Cr	Fe	Co	Ni	Cu	As	Sb	Cd	Zn	Hg	Pb	PH	TOC
Depth of 1–10 cm													
1	94.20	14,491.1	10,133.9	73.64	39	ND	0.58	ND	2.85	0.15	53.42	5.21	1.59
2	169.20	28,732.1	22,910.7	163.3	109	31.78	1.09	34.61	4.35	0.36	70.71	5.91	1.75
3	163.13	28,633.9	23,232.1	160.8	61	22.11	0.63	13.82	4.38	0.51	77.95	7.86	1.71
4	251.54	40,557.7	33,375	293.75	123	42.93	1.75	75.13	5.65	0.19	106.44	8.07	2.6
5	266.25	46,817.3	36,740.4	253.08	138	63.83	1.95	124.59	6.57	0.58	151.06	5.39	2.14
6	112.69	20,019.2	16,653.9	97.6	62	ND	0.2	ND	1.97	0.19	61.88	5.73	1.51
7	224.50	39,650	31,433.3	181	165	60.82	1.78	108.46	5.4	0.31	141.33	6.29	1.03
8	137.77	23,946.4	35,053.6	120.27	33	43.64	0.65	28.45	4.28	ND	75.77	6.57	0.63
9	124.04	21,750	22,846.2	96.92	93	1.01	0.7	0.71	4.13	ND	94.17	6.14	2.58
10	130.17	20,175	20,925	120.08	53	3.24	0.52	1.67	3.67	0.08	106.75	6.58	2.31
11	138.56	28,903.9	24,884.6	100.19	43	10.02	0.52	5.2	2.58	ND	74.23	5.86	1.14
12	149.02	29,151.8	29,098.2	122.86	61	18.28	0.71	12.89	4.03	0.08	101.07	4.79	2.23
13	88.51	15,875	10,846.2	57.9	52	ND	0.04	ND	2.75	ND	65.77	6.48	2.25
14	172.50	29,033.3	28,258.3	125.42	60	ND	0.71	ND	3.47	ND	83.05	5.76	1.71
15	110.36	23,071.4	15,116.1	70.31	36	1.3	ND	ND	2.65	ND	65.43	4.6	1.46
16	328.66	51,901.8	55,580.4	203.75	131	23.03	0.13	3.08	3.73	1.41	57.6	5.05	1.4
17	138.17	20,908.3	25,366.7	109.33	47	5.52	0.87	4.78	4.93	0.33	87.17	4.12	1.55
18	107.05	16,642.9	19,991.1	81	35	ND	0.56	ND	3.48	ND	74.8	5.33	1.68
Depth of 10–20 cm													
1	38.93	12,675	4355	28.71	13	0.69	0.22	0.15	1.13	ND	24.2	6.01	2
2	165.17	46,416.7	23,508.3	207.67	69	29.74	1.03	30.73	3.84	ND	77.34	5.89	1.85
3	95	26,758.3	13,491.7	87.08	51	15.43	0.36	5.53	2.6	ND	40.59	5.46	1.81
4	125.42	33,083.3	17,733.3	127.5	42	30.59	0.39	11.98	2.27	ND	55.28	5.66	1.5
5	145.09	36,517.9	21,232.1	138.93	57	27.24	1.13	30.65	3.06	ND	65.51	5.47	2
6	61.47	16,575	10,041.7	48.78	18	5.36	0.08	0.4	1.09	ND	30.76	6.07	1.42
7	130.63	34,928.6	20,723.2	107.23	30	34.34	0.53	18.09	2.82	0.05	94.46	5.9	0.93
8	118.75	31,000	28,875	100.8	42	44.49	0.58	25.82	3.2	0.18	79.41	5.66	0.66
9	99.13	20,884.6	17,682.7	82.69	46	ND	0.45	ND	2.54	ND	67.79	6.3	2.24
10	113.3	20,232.1	17,598.2	101.34	42	ND	0.29	ND	3.16	ND	78.18	6.42	2.17
11	70.08	17,044.6	11,857.1	65.87	12	ND	ND	0.74	1.01	ND	39.4	6.52	1.08
12	106.16	25,500	21,000	90.36	34	13.6	0.39	5.34	2.47	ND	62.59	4.4	1.94
13	65.62	13,182.7	8,005.77	49.54	57	ND	ND	2.52	2.38	ND	49.65	5.36	2.04
14	135.45	23,392.9	22,187.5	106.52	50	2.73	0.14	0.39	2.64	ND	58.49	5.35	1.99
15	93.71	19,682.7	12,990.4	59.91	23	ND	ND	0.29	1.93	ND	52.13	4.65	1.71
16	314.81	58,894.2	53,663.5	207.02	149	16.05	0.14	2.31	4.08	0.86	70.4	5.15	2.71
17	112.23	20,446.4	21,982.1	93.39	43	12.54	0.56	7.06	3.71	ND	78.78	4.28	1.46
18	146.52	25,500	26,276.8	106.52	66	0.54	1.08	0.59	4.79	0.26	96.25	5.38	1.28

S: Sample, ND: Not detected.

**Table 4:** Comparison of the median concentrations (mg/kg) result of trace elements for this study with the other studies.

Trace Elements												
Countries	Cr	Fe	Co	Ni	Cu	As	Sb	Cd	Zn	Hg	Pb	Reference
China (Wuhan)	140.10	27,304.9	22,656.94	119.12	60.73	15.58	0.58	15.44	3.32	0.15	74.16	This study
Bangladesh	-	18,000	-	24.48	20.06	8.34	-	-	-	-	0.85	[11]
Cuba	85.9	-	9.16	69.57	43.1	20.32	-	0.52	100.2	95.4	14.22	[59]
Bangladesh	53.7	30,404	-	48.1	60	4073.1	-	0.0072	209	486.6	49.66	[60]
China (Xihu district)	53.3	-	7.32	22.9	38.7	-	-	0.387	139	-	70	[8]
China (Hubei)	-	-	-	-	386	35.4	-	2.59	-	-	120	[34]
India	8.01	32.12	-	10.86	52.72	-	-	-	44.72	-	-	[61]
Tanzania	7.68	-	-	-	5.62	-	-	0.22	33.18	-	14.32	[10]
Brazil	20.61	20,273.75	7.44	12.86	111.54	-	13.81	38.31	224.29	954.88	-	[62]
Pakistan	5.86	-	7.56	22.16	18.12	-	-	0.59	-	-	16.18	[64]
Wuhan (China)	85	-	16	34	34	-	-	0.2	85	0.11	33	[32]
Wuhan (China)	152.78	-	16.37	52.87	60.85	-	-	3.98	86.4	-	30.17	[33]
Democratic republic of Congo	-	4.5	990	20	10,320	29	-	49	726	-	135	[67]
Iran	48.08	101,588.89	38.5	74.69	100.84	16	0.22	0.16	72.96	22.30	10.80	[64]
Iran	53.21	33,428.13	16.51	71.56	38.95	11.77	0.21	0.22	71.91	31.45	15.07	[64]
Soil along Chao River (China)	118	-	17.5	20	46.5	6.07	-	0.16	113	0.360	20.3	[65]
Northern Pakistan	29.94	-	36.76	26.61	35.28	-	-	2.04	101.76		4.69	[66]
Miyun Reservoir (China)	-	-	10.7–38.74	-	-	-	-	-	-	-	-	[68]

**Table 5:** Pearson’s correlation coefficient between individual trace elements and soil properties.

Elements	Cr	Fe	Co	Ni	Cu	As	Sb	Cd	Zn	Hg	Pb	pH	TOC
Cr	1												
Fe	0.961 **	1											
Co	0.920 **	0.861 **	1										
Ni	0.874 **	0.917 **	0.730 **	1									
Cu	0.904 **	0.895 **	0.750 **	0.838 **	1								
As	0.535 *	0.692 **	0.489 *	0.725 **	0.544 *	1							
Sb	0.34	0.445	0.235	0.634 **	0.426	0.733 **	1						
Cd	0.422	0.576 *	0.273	0.662 **	0.514 *	0.902 **	0.854 **	1					
Zn	0.618 **	0.609 **	0.599 **	0.719 **	0.631 **	0.635 **	0.770 **	0.609 **	1				
Hg	0.849 **	0.772 **	0.809 **	0.588 *	0.763 **	0.269	-0.003	0.1	0.363	1			
Pb	0.388	0.412	0.375	0.495 *	0.448	0.592 **	0.759**	0.694 **	0.786 **	0.022	1		
pH	−0.07	0.008	−0.165	0.173	0.07	0.222	0.139	0.202	−0.072	−0.182	0.017	1	
TOC	0.148	0.059	−0.024	0.175	0.292	−0.318	−0.048	−0.162	0.087	0.118	−0.026	0.007	1

** Correlation is significant at the 0.01 level (2-tailed); * Correlation is significant at the 0.05 level (2-tailed).

**Table 6:** Rotated component matrix of trace elements in soil.

Elements	Component	
	1	2
Cr	0.949	0.282
Fe	0.886	0.408
Co	0.904	0.198
Ni	0.737	0.579
Cu	0.846	0.377
As	0.357	0.805
Sb	0.077	0.949
Cd	0.178	0.911
Zn	0.438	0.748
Hg	0.948	−0.103
Pb	0.130	0.848
Eigenvalues % of Variance Cumulative %	7.167 65.152 65.152	2.296 20.877 86.029

Extraction Method: Principal Component Analysis. Rotation Method: Varimax with Kaiser Normalization.

**Table 7:** Contamination Factor (*C^i^_f_*), Degree of Contamination (*C_d_*), Ecological Risk Factor (*E^i^_f_*), Risk Index (*RI*), Enrichment Factor (*EF*) and Geo-accumulation Index (*I_geo_*) of trace elements.

Elements	*C^i^_f_*	*E^i^_f_*	*EF*	Degree of *EF*	*I_geo_*	Contamination Level
Cr	1.56	3.11	1.75	Low enrichment	0.05	Uncontaminated to moderately contaminated
Fe	0.93	0	1	Low enrichment	−0.69	Uncontaminated
Co	1471.23	2942.46	1584.12	Extremely high enrichment	9.94	Extremely contaminated
Ni	2.98	14.89	3.44	Moderate enrichment	0.99	Uncontaminated to moderately contaminated
Cu	1.74	8.68	2.13	Moderate enrichment	0.21	Uncontaminated to moderately contaminated
As	1.04	10.39	1.36	Low enrichment	−0.53	Uncontaminated
Sb	0.35	5.27	0.38	Low enrichment	−2.09	Uncontaminated
Cd	77.22	2316.61	90.4	Extremely high enrichment	5.69	Extremely contaminated
Zn	0.03	0.03	0.04	Low enrichment	−5.5	Uncontaminated
Hg	1	40	2.02	Moderate enrichment	−0.58	Uncontaminated
Pb	2.12	10.59	2.99	Moderate enrichment	0.5	Uncontaminated to moderately contaminated
*C_d_* = Σ*C^i^_f_* =1560.2, *RI* = Σ*E^i^_f_* = 5352.03						

## References

[1] Hashmi M.Z., Yu C., Shen H., Duan D., Shen C., Lou L. (2013). Risk Assessment of Heavy Metals Pollution in Agricultural Soils of Siling Reservoir Watershed in Zhejiang Province, China. Bio. Med. Res. Int..

[2] Lu C., Wu Y., Hu S., Zhang X., Fu Y. (2016). Distribution and Transport of Residual Lead and Copper Along Soil Profiles in a Mining Region of North China. Pedosphere.

[3] Asati A., Pichhode M., Nikhil K. (2016). Effect of Heavy Metals on Plants: An Overview. Int. J. App. Innov. Eng. Manag..

[4] Arao T., Ishikawa S., Murakami M., Abe K., Maejima Y., Makino T. (2010). Heavy metal contamination of agricultural soil and countermeasures in Japan. Paddy Water Environ..

[5] Masson P., Dalix T., Bussière S. (2010). Determination of major and trace elements in plant samples by inductively coupled plasma-mass spectrometry. Commun. Soil Sci. Plant Anal..

[6] Delang C.O. (2017). Environmental & Socio-economic Studies Causes and distribution of soil pollution in China. Environ. Socio-Econ. Studies..

[7] Xu Y., Liang X., Xu Y., Qin X., Huang Q., Wang L. (2017). Remediation of Heavy Metal-Polluted Agricultural Soils Using Clay Minerals: A. Review. Pedosphere.

[8] Wang M., Zhang H. (2018). Accumulation of Heavy Metals in Roadside Soil in Urban Area and the Related Impacting Factors. Int. J. Environ. Res. Public Health..

[9] Ma L., Yang Z., Li L., Wang L. (2016). Source identification and risk assessment of heavy metal contaminations in urban soils of Changsha, a mine-impacted city in Southern China. Environ. Sci. Pollut. Res..

[10] Kibassa D., Kimaro A.A., Shemdoe R.S. (2013). Heavy metals concentrations in selected areas used for urban agriculture in Dar es Salaam, Tanzania. Sci. Res. Essays Acad. J..

[11] Rahman M.S., Biswas P.K., Al Hasan S.M., Rahman M.M., Lee S.H., Kim K.H., Rahman S.M., Islam M.R. (2018). The occurrences of heavy metals in farmland soils and their propagation into paddy plants. Environ. Monit. Assess..

[12] Fosu-Mensah B.Y., Addae E., Yirenya-Tawiah D., Nyame F. (2017). Heavy metals concentration and distribution in soils and vegetation at Korle Lagoon area in Accra, Ghana. Cogent. Environ. Sci..

[13] Marrugo-negrete J., Pinedo-hernández J., Díez S. (2017). Assessment of heavy metal pollution, spatial distribution and origin in agricultural soils along the Sinú River Basin, Colombia. Environ. Res..

[14] Fernandes Azevedo B., Barros Furieri L., Peçanha F.M., Wiggers G.A., Frizera Vassallo P., Ronacher Simões M. (2012). Toxic Effects of Mercury on the Cardiovascular and Central Nervous Systems. J. Biomed. Biotechnol..

[15] Chauhan G., Chauhan P.U.K. (2014). Risk Assessment of Heavy Metal Toxicity Through Contaminated Vegetables From Waste. Int. J. Adv. Technol. Eng. Sci..

[16] Olatunji O.S., Opeolu B.O., Fatoki O.S., Ximba B.J. (2013). Heavy metals concentration levels in selected arable agricultural soils in South Western Nigeria. Int. J. Phys. Sci..

[17] Chang C.Y., Yu H.Y., Chen J.J., Li F.B., Zhang H.H., Liu C.P. (2014). Accumulation of heavy metals in leaf vegetables from agricultural soils and associated potential health risks in the Pearl River Delta, South China. Environ. Monit. Assess..

[18] Tangahu B.V., Sheikh Abdullah S.R., Basri H., Idris M., Anuar N., Mukhlisin M. (2011). A review on heavy metals (As, Pb, and Hg) uptake by plants through phytoremediation. Int. J. Chem. Eng..

[19] Yuan G.L., Sun T.H., Han P., Li J., Lang X.X. (2014). Source identification and ecological risk assessment of heavy metals in topsoil using environmental geochemical mapping: Typical urban renewal area in Beijing, China. J. Geochem. Explor..

[20] Hu H., Jin Q., Kavan P. (2014). A Study of Heavy Metal Pollution in China: Current Status, Pollution-control Policies and Countermeasures. Sustainability.

[21] Yu H., Wang J., Fang W., Yuan J., Yang Z. (2006). Cadmium accumulation in different rice cultivars and screening for pollution-safe cultivars of rice. Sci. Total Environ..

[22] Boke A., Megersa N., Teju E. (2015). Quantitative Determination of the Heavy Metal Levels in the Wild Edible Plant Parts and their Corresponding Soils of the Central and Western Regions of the Oromia State, Ethiopia. J. Environ. Anal. Toxicol..

[23] Kim R.Y., Yoon J.K., Kim T.S., Yang J.E., Owens G., Kim K.R. (2015). Bioavailability of heavy metals in soils: Definitions and practical implementation—A critical review. Environ. Geochem. Health.

[24] Ogunlaja O.O.O., Moodley R., Baijnath H., Jonnalagadda S.B. (2017). Nutritional evaluation, bioaccumulation and toxicological assessment of heavy metals in edible fruits of FicussurForssk (Moraceae). J. Environ. Sci. Health.

[25] Hussain A., Abbas N., Arshad F., Akram M., Khan Z.I., Ahmad K. (2013). Effects of diverse doses of Lead (Pb) on different growth attributes of *Zea-Mays* L.. Agric. Sci..

[26] Barrachina A.C., Carbonell F.B., Beneyto J.M. (1995). Arsenic uptake, distribution, and accumulation in tomato plants: Effect of arsenite on plant growth and yield. J. Plant Nutr..

[27] Kamunda C., Mathuthu M., Madhuku M. (2016). Health risk assessment of heavy metals in soils from witwatersrand gold mining basin, South Africa. Int. J. Environ. Res. Public Health.

[28] Wang M.Y., Chen A.K., Wong M.H., Qiu R.L., Cheng H., Ye Z.H. (2011). Cadmium accumulation in and tolerance of rice (*Oryza sativa* L.) varieties with different rates of radial oxygen loss. Environ. Pollut..

[29] Lin Y.S., Caffrey J.L., Lin J.W., Bayliss D., Faramawi M.F., Bateson T.F. (2013). Increased risk of cancer mortality associated with cadmium exposures in Older Americans with low Zinc Intake. J. Toxicol. Environ. Health.

[30] Nieboer E., Tsuji L.J.S., Martin I.D., Liberda E.N. (2013). Human biomonitoring issues related to lead exposure. Environ. Sci. Process. Impacts.

[31] Hagberg A. (2007). Industrial Wastewater Treatment and other Environmental Problems in Wuhan—Is Swedish Technology a Solution?.

[32] Gong M., Wu L., Bi X.Y., Ren L.M., Wang L., Ma Z.D., Bao Z.Y., Li Z.G. (2010). Assessing heavy-metal contamination and sources by GIS-based approach and multivariate analysis of urban-rural topsoils in Wuhan, central China. Environ. Geochem. Health.

[33] Qu M.K., Li W.D., Zhang C.R., Wang S.Q., Yang Y., He L.Y. (2013). Source Apportionment of Heavy Metals in Soils Using Multivariate Statistics and Geostatistics. Pedosphere.

[34] Li Z., Zhang G., Liu Y., Wan K.Y., Zhang R., Chen F. (2013). Soil Nutrient Assessment for Urban Ecosystems in Hubei, China. PLoS ONE.

[35] Lv J., Ma T., Dong Z., Yao Y. (2018). Temporal and Spatial Analyses of the Landscape Pattern of Wuhan City Based on Remote Sensing Images. Int. J. Geo-Inf..

[36] Sekabira K., Oryem Origa H., Basamba T.A., Mutumba G., Kakudidi E. (2010). Assessment of heavy metal pollution in the urban stream sediments and its tributaries. Int. J. Environ. Sci. Tech..

[37] Makokha V.A., Qi Y., Shen Y., Wang J. (2016). Concentrations, Distribution, and Ecological Risk Assessment of Heavy Metals in the East Dongting and Honghu Lake, China. Expo. Health.

[38] Weldegebriel Y., Chandravanshi B.S., Wondimu T. (2012). Concentration levels of metals in vegetables grown in soils irrigated with river water in Addis Ababa, Ethiopia. Ecotoxicol. Environ. Saf..

[39] Wlakey A. (1947). A critical examination of a rapid method for determination of organic carbon in soils—Effect of variations in digestion conditions and of inorganic soil constituents. Soil Sci..

[40] Liu H., Liu G., Zhou C., Yuan Z., Da C. (2017). Geochemical speciation and ecological risk assessment of heavy metals in surface soils collected from the Yellow River Delta National Nature Reserve, China. Hum. Ecol. Risk Assess..

[41] Hakanson L. (1980). An ecological risk index for aquatic pollution control.a sedimentological approach. Water Res..

[42] Yang Z., Wang Y., Shen Z., Niu J., Tang Z. (2009). Distribution and speciation of heavy metals in sediments from the mainstream, tributaries, and lakes of the Yangtze River catchment of Wuhan, China. J. Hazard Mater..

[43] He M., Wang X., Wu F., Fu Z. (2012). Antimony pollution in China. Sci. Total Environ..

[44] Jiao X., Teng Y., Zhan Y., Wu J., Lin X. (2015). Soil heavy metal pollution and risk assessment in Shenyang industrial district, Northeast China. PLoS ONE.

[45] Muller H.G. (1969). Mechanical Properties, Rheology, and Haptaesthesis of Food. J. Texture Stud..

[46] Ogundiran M.B., Osibanjo O. (2009). Mobility and speciation of heavy metals in soils impacted by hazardous waste. Chem. Speciat. Bioavailab..

[47] Liu L., Lu J., Zhang Z., Zheng H., Gao X., Zhang W. (2014). Heavy Metals Contamination in Greenhouse Soils and Vegetables in Guanzhong, China. J. Encapsulation Adsorption Sci..

[48] Bai Y., Wang M., Peng C., Alatalo J.M. (2016). Impacts of urbanization on the distribution of heavy metals in soils along the Huangpu River, the drinking water source for Shanghai. Environ. Sci. Pollut. Res..

[49] Jiang X., Lu W.X., Zhao H.Q., Yang Q.C., Yang Z.P. (2014). Potential ecological risk assessment and prediction of soil heavy-metal pollution around coal gangue dump. Nat. Hazards Earth Syst. Sci..

[50] Fang C., Moncrieff J.B. (2005). The variation of soil microbial respiration with depth in relation to soil carbon composition. Plant Soil.

[51] Celik I. (2005). Land-use effects on organic matter and physical properties of soil in a southern Mediterranean highland of Turkey. Soil Tillage Res..

[52] Behbahaninia A., Mirbagheri S.A., Khorasani N., Nouri J., Javid A.H. (2009). Heavy metal contamination of municipal effluent in soil and plants. J. Food Agric. Environ..

[53] Rahaman A., Sadia Afroze J., Bashar K., Farhad Ali M., Razib Hosen M. (2016). A Comparative Study of Heavy Metal Concentration in Different Layers of Tannery Vicinity Soil and Near Agricultural Soil. Am. J. Anal. Chem..

[54] Li J., Lu Y., Yin W., Gan H., Zhang C., Deng X., Lian J. (2009). Distribution of heavy metals in agricultural soils near a petrochemical complex in Guangzhou, China. Environ. Monit. Assess..

[55] Camobreco V.J., Richards B.K., Steenhuis T.S., Peverly J.H., McBride M.B. (1996). Movement of heavy metals through undisturbed and homogenized soil columns. Soil Sci..

[56] Akenga T., Sudoi V., Machuka W., Kerich E. (2016). Heavy Metal Concentrations in Agricultural Farms in Homa Hills Homa Bay County, Kenya. Int. J. Sci. Res..

[57] Yabe J., Ishizuka M., Umemura T. (2010). Current Levels of Heavy Metal Pollution in Africa. J. Vet. Med. Sci..

[58] Mungai T.M., Owino A.A., Makokha V.A., Gao Y., Yan X., Wang J. (2016). Occurrences and toxicological risk assessment of eight heavy metals in agricultural soils from Kenya, Eastern Africa. Environ. Sci. Pollut. Res..

[59] Accioly A., Montero A., Ugarte O.M., do Nascimento C.W., de Aguiar Accioly A.M., Biondi C.M., da Silva Y.J. (2015). Background concentrations and reference values for heavy metals in soils of Cuba. Environ. Monit. Assess..

[60] Rahman S.H., Khanam D., Adyel T.M., Islam M.S., Ahsan M.A., Akbor M.A. (2012). Assessment of Heavy Metal Contamination of Agricultural Soil around Dhaka Export Processing Zone (DEPZ), Bangladesh: Implication of Seasonal Variation and Indices. Appl. Sci..

[61] Rakesh Sharma M.S., Raju N.S. (2013). Correlation of Heavy Metal contamination with Soil properties of Industrial areas of Mysore, Karnataka, India by Cluster analysis. Int. Res. J. Environ. Sci..

[62] De Almeida Júnior A.B., do Nascimento C.W.A., Biondi C.M., de Souza A.P., Barros F.M., do Rêgo Barros F.M. (2016). Background and reference values of metals in soils from Paraíba state, Brazil. Rev. Bras. Cienc. Sol..

[63] Tariq S.R., Shafiq M., Chotana G.A. (2016). Distribution of Heavy Metals in the Soils Associated with the Commonly Used Pesticides in Cotton Fields. Scientifica.

[64] Soltani N., Keshavarzi B., Moore F., Sorooshian A., Ahmadi M.R. (2017). Distribution of potentially toxic elements (PTEs) in tailings, soils, and plants around Gol-E.-Gohar iron mine, a case study in Iran. Environ. Sci. Pollut. Res..

[65] Qin F., Ji H., Li Q., Guo X., Tang L., Feng J. (2014). Evaluation of trace elements and identification of pollution sources in particle size fractions of soil from iron ore areas along the Chao River. J. Geochem. Explor..

[66] Rehman I.U., Ishaq M., Ali L., Khan S., Ahmad I., Din I.U. (2018). Enrichment, spatial distribution of potential ecological and human health risk assessment via toxic metals in soil and surface water ingestion in the vicinity of Sewakht mines, district Chitral, Northern Pakistan. Ecotoxicol. Environ. Saf..

[67] Pourret O., Lange B., Bonhoure J., Colinet G., Decrée S., Mahy G., Séleck M., Shutcha M., Faucon M.-P. (2016). Assessment of soil metal distribution and environmental impact of mining in Katanga (Democratic Republic of Congo). Appl. Geochem..

[68] Gao L., Gao B., Zhou Y., Xu D., Sun K. (2017). Predicting remobilization characteristics of cobalt in riparian soils in the Miyun Reservoir prior to water retention. Ecol. Indic..

[69] Khaledian Y., Pereira P., Brevik E.C., Pundyte N., Paliulis D. (2017). The Influence of Organic Carbon and pH on Heavy Metals, Potassium, and Magnesium Levels in Lithuanian Podzols. Land Degrad. Dev..

[70] Constantin C. (2014). Principal Component Analysis—A Powerful Tool in Computing Marketing Information. Bull. Transilv. Univ. Brasov Ser. V Econ. Sci..

[71] Yang J., Teng Y., Song L., Zuo R. (2016). Tracing sources and contamination assessments of heavy metals in road and foliar dusts in a typical mining city, China. PLoS ONE.

[72] Abdulqaderismaeel W., Kusag A. (2015). Enrichment Factor and Geo-accumulation Index for Heavy Metals at Industrial Zone in Iraq. IOSR J. Appl. Geol. Geophys..

[73] Zhang Z., Juying L., Mamat Z. (2016). Sources identification and pollution evaluation of heavy metals in the surface sediments of Bortala River, Northwest China. Ecotoxicol. Environ. Saf..

[74] Gupta S., Jena V., Matic N., Kapralova V., Solanki J.S. (2014). Assessment of Geo-Accumulation Index of Heavy Metal and Source of Contamination By Multivariate Factor Analysis. Int. J. Hazard Mater..

[75] Alshahri F., El-Taher A. (2018). Assessment of Heavy and Trace Metals in Surface Soil Nearby an Oil Refinery, Assessment of Heavy and Trace Metals in Surface Soil Nearby an Oil Refinery, Saudi Arabia, Using Geoaccumulation and Pollution Indices. Arch. Environ. Contam. Toxicol..

